# Cellular Aspects of Prion Replication *In Vitro*

**DOI:** 10.3390/v5010374

**Published:** 2013-01-22

**Authors:** Andrea Grassmann, Hanna Wolf, Julia Hofmann, James Graham, Ina Vorberg

**Affiliations:** 1 German Center for Neurodegenerative Diseases (DZNE e.V.), Ludwig-Erhard-Allee 2, 53175 Bonn, Germany; E-Mails: andrea.grassmann@dzne.de (A.G.); hanna.wolf@dzne.de (H.W.); julia.hofmann@dzne.de (J.H.); james.graham@dzne.de (J.G.); 2 Rheinische Friedrich-Wilhelms-Universität Bonn, Bonn, Germany

**Keywords:** prion, prion strains, transmissible spongiform encephalopathies, glycosaminoglycans, LRP1, RPSA

## Abstract

Prion diseases or transmissible spongiform encephalopathies (TSEs) are fatal neurodegenerative disorders in mammals that are caused by unconventional agents predominantly composed of aggregated misfolded prion protein (PrP). Prions self-propagate by recruitment of host-encoded PrP into highly ordered β-sheet rich aggregates. Prion strains differ in their clinical, pathological and biochemical characteristics and are likely to be the consequence of distinct abnormal prion protein conformers that stably replicate their alternate states in the host cell. Understanding prion cell biology is fundamental for identifying potential drug targets for disease intervention. The development of permissive cell culture models has greatly enhanced our knowledge on entry, propagation and dissemination of TSE agents. However, despite extensive research, the precise mechanism of prion infection and potential strain effects remain enigmatic. This review summarizes our current knowledge of the cell biology and propagation of prions derived from cell culture experiments. We discuss recent findings on the trafficking of cellular and pathologic PrP, the potential sites of abnormal prion protein synthesis and potential co-factors involved in prion entry and propagation.

## 1. Introduction

Prion diseases or transmissible spongiform encephalopathies (TSEs) are neurodegenerative disorders that affect many mammalian species. TSEs include Creutzfeldt-Jakob disease, fatal familial insomnia and Gerstmann-Sträussler-Scheinker syndrome in humans, scrapie in sheep and goats, chronic wasting disease in deer and elk and bovine spongiform encephalopathy. In humans, prion diseases can be sporadic, infectious or of genetic origin. Natural genetic prion diseases in animals have not been reported until now. In animals, infection occurs mainly through the intestinal tract due to ingestion of prions present in the food or the natural environment [1]. During TSE disease, an abnormally folded conformer (PrP^Sc^) of the cellular prion protein (PrP^C^) accumulates in the central nervous and lymphoreticular system of the infected host. According to the prion hypothesis, PrP^Sc^ constitutes the major, if not only, component of the proteinaceous infectious particles [2,3]. The conversion of the host-encoded PrP^C^ to PrP^Sc ^is a post-translational process that involves a conformational change from a predominantly α-helical structure to a protein fold increased in ß-sheet. PrP^Sc^ is likely generated by a seeded polymerization reaction in which it serves as a template that binds to normal PrP^C^ and catalyzes its conformational conversion to an abnormal, aggregated isoform. PrP aggregates consist of fibrils with a cross-ß-structure that is characteristic of amyloid. As the amyloid fibril elongates and matures, it acquires an increase in conformational stability that is resistant to denaturation by heating, detergents and proteases. Amyloid fibrils are associated with many other neurodegenerative protein misfolding disorders, notably Alzheimer’s and Parkinson’s disease [4]. However, prion diseases are unconventional protein misfolding disorders because they constitute infectious diseases that are often naturally transmitted within species and sometimes even across species barriers. 

The first prion disease studied was scrapie of sheep and goats. Seminal work on scrapie by Pattison and Millson in 1961 laid the foundations for the hypothesis that prions exist as different strains [5]. At least 20 different prion strains have been isolated from scrapie that can be propagated in the same inbred mouse line. Prion strains are distinguished by several semi-quantitative factors including incubation time before disease onset, lesion profiles in the brain and the areas of deposition of aggregated PrP [6,7]. PrP^Sc^ molecules associated with prion strains differ in their biochemical and biophysical properties. For example, PrP^Sc^ molecules exhibit strain-specific glycosylation profiles, and differ in their resistance to proteases as well as in their binding to conformation-specific antibodies [8,9]. This led to the proposal that prion strains are enciphered by the specific fold of PrP^Sc^ [3,10]. According to this theory, strain-specific PrP^Sc^ conformations would be adopted and amplified by the binding and subsequent conversion of PrP^C^, thereby preserving the strain-specific information enciphered by the respective quaternary structures of PrP^Sc^.

## 2. The Cellular Prion Protein PrP^C^: Structure, Biosynthesis and Intracellular Trafficking

In 1985 researchers identified the *Prnp* gene encoding the prion protein [11,12] on chromosome 20 in humans and chromosome 2 in mice [13,14]. The *Prnp* gene is evolutionary highly conserved, exhibiting a sequence homology of approximately 80% from amphibia to mammals [15,16,17]. The *Prnp* gene contains two to three exons depending on the species, with the last exon encoding the open reading frame [12]. Cellular prion protein is constitutively expressed in many tissues, including the central and peripheral nervous system as well as the immune, lymphoreticular and intestinal system [18]. A particularly high expression is found in neurons localized both at pre- and post-synaptic sites [19] and in glial cells [20].

**Figure 1:**
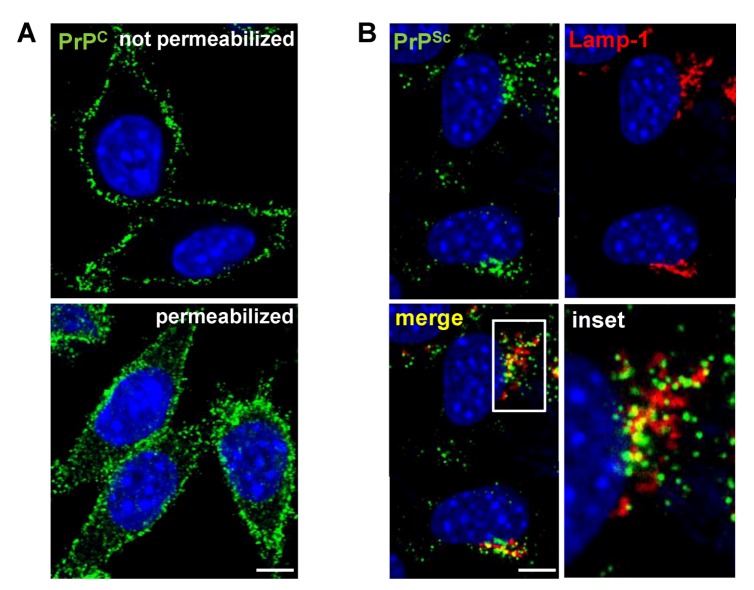
Localization of PrP^C^ and PrP^Sc^ in L929 fibroblast cells. (**A**) Indirect immunofluorescence (IF) staining of cellular PrP (green) in uninfected L929 cells. PrP^C^ predominantly resides at the cell surface with some intracellular localization. (**B**) Detection of PrP^Sc^ in L929 cells persistently infected with prion strain 22L by IF. In contrast to PrP^C^, PrP^Sc^ (green) primarily localizes intracellularly and partially co-localizes with the lysosomal marker Lamp-1 (red). (**A,B**) Nuclei were counterstained with Hoechst (blue). Scale bar: 5 µm.

PrP^C^ is synthesized on the rough endoplasmic reticulum (ER) and transits through the Golgi apparatus to the cell surface (Figure 1A). Within the ER and Golgi, PrP^C^ becomes glycosylated at two asparagine residues [21]. Further post-translational modifications include the formation of a disulfide bond between two cysteine residues (amino acid residues 179 and 214 in human PrP) [22] and the attachment of a glycosyl-phosphatidyl-inositol (GPI) moiety at the carboxy-terminus of the protein [23]. At the plasma membrane, PrP^C^ is incorporated into lipid rafts and caveolae (raft structures with caveolin-1), which are regions of the membrane enriched in cholesterol and sphingolipids [24,25]. Targeting to these lipid rafts is mediated by the amino-terminus of PrP^C^ [26,27]. An early association of PrP^C^ with lipid rafts during its biosynthesis appears to be necessary for its correct folding [28]. Although PrP^C^ is normally translocated to the plasma membrane, high concentrations have been detected within multivesicular bodies [29]. Once on the plasma membrane, PrP^C^ can undergo proteolytic processing by metalloproteases, resulting in a membrane-attached carboxyterminal (C1) and an extracellularly released amino-terminal fragment [30,31,32,33]. In addition, it has been observed that a small percentage of full-length PrP^C^ molecules is secreted, either in a soluble form [34,35] or in association with exosomes [36,37]. Within the cell there is a minor sub-population of PrP^C^ molecules present in the cytosol [38]. Interestingly, using an inducible cell line, PrP23-230 was found in the nucleus of these cells and in association with chromatin [39]. The physiological relevance of such intranuclear localization so far is unclear.

Extensive research into the biological function of PrP^C^ has resulted in a plethora of different possible functions. So far, these include involvement in signaling cascades, neuronal survival, apoptosis, oxidative stress, cell adhesion, differentiation, immunomodulation and more recently, microRNA metabolism [40,41]. PrP^C ^has a high affinity for metals such as copper, zinc and manganese through binding at its amino-terminus. Binding to PrP^C^ mediates neuronal uptake of these metal ions potentially through interaction with other receptors [42,43]. PrP^C^ has also been proposed to act as a cell surface scaffold protein that interacts with different partners. These mediate the activation of a range of diverse signaling pathways that modulate neuritogenesis and synapse formation [40]. Interactions of PrP^C ^with the neural cell adhesion molecule NCAM or with the laminin receptor precursor LRP/LR have been reported to elicit specific signaling cascades in neurons [44,45,46]. In non-neuronal cells, PrP^C^ also plays an important role during embryogenesis or during stem-cell proliferation and differentiation [47,48]. Interestingly, PrP has also been shown to bind both RNA and DNA *in vitro *[49,50,51,52]. Evidence for a physiological role of these nucleic acid-protein associations [41] is accumulating but needs further clarification.

PrP^C^ is rapidly and constitutively endocytosed from the plasma membrane [53,54]. External stimuli such as the binding of copper or stress-inducible protein 1 (STI1) to PrP^C^ can stimulate its internalization [55]. Endocytosis occurs via a dynamin-dependent pathway. PrP^C^ transits through Rab5 positive early endosomes (EEs) before it is degraded via the endosomal/lysosomal pathway [54,56,57,58,59]. Alternatively, endocytosed PrP^C^ can transfer rapidly and directly to recycling endosomes (RE) and back to the cell surface [54,56,60,61]. It has been proposed that the dynamin-dependent endocytosis of PrP^C^ is a GPI-anchor independent event mediated by the interaction of other proteins with specific domains within PrP^C^ [56].

**Figure 2:**
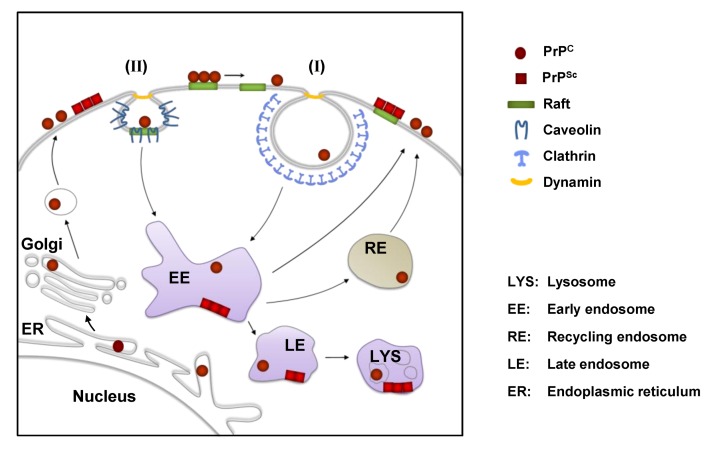
Cell biology of PrP in scrapie-infected cells. PrP^C^ is synthesized in the endoplasmic reticulum (ER) and passes through the secretory pathway to the cell surface, where it resides in lipid rafts. In many cells, PrP^C^ leaves lipid rafts prior to being internalized by clathrin-dependent endocytosis (I). Clathrin-independent raft/caveolae-dependent internalization (II) of PrP^C^ has also been proposed for some cells. PrP^C^ can be degraded by lysosomes or rapidly recycled back to the cell surface by recycling endosomes (RE). In cultured scrapie-infected cells the conversion of PrP^C^ to PrP^Sc^ is believed to take place on the cell surface and/or in vesicles along the endolysosomal pathway. After conversion PrP^Sc^ can accumulate at the cell surface or in intracellular vesicles (e.g. lysosomes).

Both, clathrin-dependent and -independent pathways have been described for PrP^C^ internalization [54,59,62,63,64] (Figure 2). Although PrP^C^ may be endocytosed through rafts in some cells [62,64,65], most studies demonstrate that PrP^C^ translocates out of rafts prior to its internalization via clathrin-coated pits in permanent cell cultures and primary neurons [54,60,66,67,68,69]. An amino-terminal, positively charged domain of PrP^C^ is important for its endocytosis by clathrin-coated vesicles [54,66]. PrP^C^ has been detected in clathrin-coated vesicles using electron microscopy [54,60,70]. Still, PrP^C^ internalization in mature primary hippocampal neurons appears to depend on rafts and cholesterol [71]. In agreement with this, Sarnataro *et al.* showed that lipid rafts and clathrin-coated vesicles can co-operate in the internalization of PrP^C^ [72]. The conflicting results obtained in different cell culture models argue that the internalization of PrP^C^ is a complex event that may involve different receptors and co-receptors and more than one endocytic route depending on the cell type or stimulus. 

## 3. Cell Surface Receptors for PrP^C^

Clathrin-coated vesicles mediate internalization of transmembrane proteins by interaction with accessory proteins [73]. Since PrP^C^ lacks a transmembrane domain capable of interacting with adaptor proteins, co-internalization of PrP^C^ with other proteins has been suggested. Several potential receptors for co-internalization have been proposed, including the laminin receptor precursor LRP/LR, the low-density lipoprotein receptor-related protein 1 (LRP1) and glycosaminoglycans (GAGs). Comparative studies on the involvement of these receptors for PrP^C^ endocytosis are lacking, so it is still unclear which role these receptors play in certain cell types. Of note, also other so far unidentified receptors might be involved. 

The membrane-associated form of the ribosomal protein SA (RPSA), termed laminin receptor precursor LRP/LR, has been characterized as a potential binding partner of PrP [74,75,76,77]. RPSA is a multifunctional protein present on the cell surface or associated with cytosolic ribosomes. The 67 kDa membrane-bound form LRP/LR is a high affinity receptor for laminin derived from a 37 kDa polypeptide (37LRP) precursor by homo- or heterodimerization through fatty acid acylation. LRP/LR is expressed in a variety of tissues and cells, including neurons [78], and also binds growth factors, toxins and pathogens. LRP/LR was first identified in a yeast two-hybrid system using a HeLa cDNA library as prey and PrP as bait [76]. Interaction of PrP23-231 and LRP/LR, both ectopically expressed, was confirmed in insect cells and N2a cells. Recombinant human PrP also bound to N2a and BHK cells expressing LRP/LR [74]. Direct binding of recombinant PrP to LRP/LR is mediated through a region in PrP encompassing amino-acid residues 144-179 in human PrP [77]. Recombinant PrP also indirectly associates with LRP/LR on the surface of CHO cells through the interaction of both molecules with the GAG heparan sulfate proteoglycan (HSPG). Although an association of PrP and LRP/LR was confirmed in an interactome analysis of tagged-PrP expressed in neuroblastoma cells, co-internalization of LRP/LR and endogenously expressed GPI-anchored PrP^C^ has not been formally demonstrated [79]. 

Another putative PrP^C^ co-receptor, LRP1, belongs to the low-density lipoprotein (LDL) receptor family and is abundantly expressed in neurons and hepatocytes [80]. LRP1 acts as a scavenger receptor with two clusters of complement-type repeats with high affinity for at least 24 different ligands [80]. Ligands either directly adhere to LRP1 or initially bind to HSPG before being scavenged by LRP1 for endocytosis. Thus, HSPGs serve as a primary docking site for ligands acting as a ligand reservoir and thereby regulating LRP1 activity. Recognition sites for cytosolic adaptor proteins in the cytoplasmic tail of the light chain regulate receptor-mediated endocytosis by clathrin-coated pits (CCPs) [80]. LRP1 transiently associates with rafts before it undergoes rapid endocytosis by CCPs. Partial lipid raft localization has been demonstrated in some, but not all cell lines tested [81]. LDL receptor family members also physically and functionally interact with other cell surface proteins, including GPI-anchored proteins and thereby modulate their activity [80]. Bridging of LRP1 to other cell surface receptors by ligands or cytosolic adaptor proteins has been shown to promote their co-internalization [81]. PrP^C^ and LRP1 have been shown to co-localize on the cell surface of SH-SY5Y cells [82]. In neurons, association of PrP^C^ with LRP1 in the secretory pathway assists in trafficking of PrP^C^ to the cell membrane [83]. Both, knock-down of LRP1 or the use of LRP antagonists, inhibit endocytosis of PrP^C^ [82,83]. A stretch of basic amino acid residues in the amino-terminus of PrP^C^ mediates binding to LRP1. HSPGs have been shown to be required for LRP1-protein complex internalization in some cases [84]. 

The metabolism and trafficking of PrP^C^ is influenced by the interaction with cell surface bound GAGs. These are long unbranched polysaccharides, composed of repeating disaccharide units that are highly sulfated. GAGs are either secreted or linked to core proteins to make an entity known as a proteoglycan. Proteoglycans are abundantly expressed on cell surfaces and differ by their polysaccharide backbone and the degree of sulfation. Heparin is a GAG mainly produced and secreted by mast cells that is structurally closely related to heparan sulfate (HS). The most common disaccharide unit within HS consists of glucuronic acid and N-acetylglucosamine. HS side chains are covalently linked to either transmembrane syndecans or GPI-anchored glypicans. The basic amino acid motif KKRPKP present in the amino-terminus of PrP (residues 23-28) and two additional regions (residues 53-93 and 110-120) are important for the association of PrP^C^ with HS [85,86,87]. Experimentally, brain-derived PrP^C^ as well as recombinant PrP have been shown to bind to heparin or another polyanion, Congo red, *in vitro* [88,89]. Recombinant PrP has also been shown to bind to HSPG on the plasma membrane of CHO cells [90].

Whether HSPGs are important for the cell surface localization of PrP^C^ remains controversial. In N2a cells, degradation of HS by heparinases neither affected the cell surface expression of PrP^C^ nor influenced its raft association [91]. GAG mimetics could potentially modulate cell surface expression of PrP^C^ by competing for the binding site of endogenously expressed HSPGs, as exogenously added soluble GAGs have been previously shown to influence cellular PrP^C^ levels. Early studies demonstrated that treatment of N2a cells with HS increased total cellular levels of PrP^C^ [88]. While pentosan polysulfate (PPS), a GAG analogue, had no apparent effect on PrP^C^ cell surface expression in N2a cells at concentrations of 0.1 μg/mL [89], a concentration of 100 μg/mL drastically reduced PrP^C^ cell membrane localization [90]. Likewise, other polyanionic compounds such as Congo red and dextran sulfate 500 kDa (DS500) at concentrations of 10 μg/mL reduced the amount of cell surface PrP^C^ in N2a cells [90]. PPS treatment did not affect biosynthesis or trafficking through the secretory pathway but instead enhanced the endocytosis rate of PrP^C^, resulting in a redistribution of a proportion of PrP^C^ into late endosomal vesicles. The amino-terminus, comprising residues 25-91, was shown to be important for this. In contrast, GAG analogue suramin was shown to impair PrP^C^ folding in the secretory pathway, resulting in the re-routing of PrP^C^ to acidic compartments [92]. 

## 4. Cellular Models for Studying PrP^Sc^ Formation

Cell culture models replicating prion infectivity were already established in 1970 [93], even before PrP^Sc^ was identified as a surrogate marker and potential TSE agent. PrP^Sc^ formation was first detected in the murine neuroblastoma cell line N2a when exposed to mouse-adapted scrapie [94,95]. Subsequent infection experiments demonstrated susceptibility of N2a cells to several different mouse-adapted scrapie strains [96]. Since then, several cell lines of neuronal and non-neuronal origin have been identified to be susceptible to a stable infection with prions (Table 1). Once prions have successfully infected a cell line, they can replicate persistently over multiple cell passages, with very few exceptions [97], without any overt cytopathic effect. Cell lines that have been successfully infected include microglial cells as well as epithelial cells, fibroblasts and myoblasts, which have all been demonstrated to persistently replicate an array of prion strains *in vitro* [98,99,100,101,102,103,104,105,106,107,108]. Curiously, a rabbit kidney epithelial cell line genetically engineered to express PrP^C^ of different species was shown to be susceptible to a variety of prion strains isolated from different sources [99,100,101,102,103,104,105,109]. Several primary cell culture models for prion replication have been reported, some of which show cytopathic effects upon infection [110,111,112,113,114,115].

Despite recent success with prion cell culture models (Table 1), prion infection of cells *in vitro* has been notoriously difficult and often unsuccessful. Most cell lines expressing PrP^C^ are resistant to prion infection, and for many prion strains, suitable cell culture models have not been established [95,116,117,118]. Importantly, *ex vivo* models for the propagation of prion strains of human origin have only been reported once [119]. Infections with human strains were more successful when prions had been previously adapted to mice [99,120]. Whilst expression of PrP^C^ is necessary for prion infection *in vitro* [121,122], the expression level of PrP^C^ does not generally appear to influence susceptibility [107,118,123]. Importantly, infection rates and prion titers in cell culture are usually low and subsequent cloning of infected cells or pre-selection of clones is a necessary process to increase the percentage of infected cells within a cell population [95,118,124,125,126,127]. Remarkably, persistent prion infection is often lost over continuous passage. Changes in growth medium composition and culture conditions can account for prion loss in cell culture [128,129]. Additionally, genetic heterogeneity and chromosomal instability have been proposed to affect susceptibility of cell populations over time [118,130]. 

Cell lines that are susceptible to some prion strains demonstrate a remarkable resistance to other strains [107,118,125]. The mouse derived fibroblast cell line NIH/3T3 for example has been shown to be susceptible only to mouse-adapted scrapie strain 22L, whilst the murine fibroblast cell line L929 is capable of replicating the strains 22L, RML and ME7 [107,125]. The reason for the differences in susceptibility to prion infection is unclear but points to substantial differences in the cell biology of prion strain replication. So far, susceptibility of a cell line to any given prion strain can only be determined empirically.

A major restriction in the analysis and understanding of prion cell biology is the specific detection of the disease-associated isoform PrP^Sc^ over the host-encoded isoform. There is a shortage of antibodies that are suitable for the convincing and specific detection of PrP^Sc^ by western blot or immunofluorescence. Therefore, it is extremely difficult to investigate the uptake of PrP^Sc^, the subcellular distribution and location of *de novo* synthesis. Presently, the protocols for the specific detection of the misfolded isoform take advantage of the unique biochemical features of PrP^Sc^ and include treatments with denaturants to enhance immunoreactivity [131]. Moreover, newly generated PrP^Sc^ cannot be discriminated from the inoculated PrP^Sc^ used, unless either the substrate PrP^C^ or template PrP^Sc^ are tagged by antibody-specific epitopes or fluorescent labels. In most studies done so far, cells overexpressed tagged PrP^C^ [132,133,134]. Thus, either the presence of the tag or overexpression of PrP^C^ could influence the conversion process. Amino-acid residue substitutions in PrP often create complications such as a transmission barrier. Tagging of PrP^C^ at the amino-terminus with GFP has been shown to compromise prion infection and PrP^Sc^ formation *in vivo* and *in vitro* [135]. Alternatively, fluorescent labeling of purified prion preparations has been successfully used to study prion uptake and intraneuronal transport *in vitro*. However, the uptake characteristics of labeled fibrils show striking differences compared to those of untagged PrP^Sc^ from crude brain homogenate preparations [133,136]. More recently, 3F4-tagged PrP^Sc^ derived from transgenic mice that were infected with prions proved effective in studying prion uptake [133]. Of note, changes in the PrP amino acid substitutions could affect prion strain characteristics and might thus not be suited to study the cell biology of different prion strains.

Previously, prion cell culture systems relied on the detection of PrP^Sc^ as a marker for infection and prion titers were determined by inoculation of cell lysates into panels of mice [107]. A major breakthrough came in determining the titers of standard prion strains with the development of the “Standard Scrapie Cell Assay” (SSCA) [118]. The SSCA incorporates a highly susceptible N2a subclone that is inoculated with serial dilutions of the prion strain RML as a standard. These infected N2a cells are propagated in a microtiter format until *de novo* formed PrP^Sc^ accumulates to detectable levels. After three cell passages, defined numbers of cells are filtered onto nitrocellulose membranes and PrP^Sc^ positive cells are detected by immunoblot using an ELISPOT reader. The SSCA can also be used as an endpoint assay (SCEPA) to quantify prion titers of individual samples by comparison with the standard titration curve [137]. The SSCA was subsequently adapted to a panel of cell lines exhibiting selective susceptibility to different strains [125,138].

**Table 1:** Cell culture models susceptible to transmissible spongiform encephalopathy (TSE) agents.

Cell designation	Tissue of origin or cell type	Species of origin	Prion strain	References
**1. Neuronal or brain-derived cells**				
N2a	neuroblastoma cell line*	mouse	Chandler, RML, 139A, 22L, C506, Fukuoka-1, FU CJD	[95,96,127,139,140,141,142,143,144]
GT1	hypothalamic cell line	mouse	Chandler, RML, 139A, 22L, kCJD, FU CJD , M1000	[96,97,99,120,139,145]
SN56	cholinergic septal cell line	mouse	Chandler, ME7, 22L	[146]
HpL3-4	hippocampal PrP-deficient cell line, upon ectopic expression of moPrP*	mouse	22L	[121,147]
CF10	brain derived PrP-deficient cell line, upon ectopic expression of moPrP	mouse	22L	[122]
SMB	prion-infected brain cell	mouse	Chandler, 139A, 22F, 79A	[93,148,149]
CAD	catecholaminergic cell line	mouse	RML, 22L, 22F, 79A, 139A, ME7	[125,150,151,152]
MG20	microglial cell line overexpressing PrP^C^	tg20 mouse	Chandler, ME7, Obihiro, mouse-adapted BSE	[98]
PC12	pheochromocytoma cell line	rat	139A, ME7	[153,154,155]
HaB	brain-derived cell line	hamster	Sc237	[131]
SH-SY5Y	neuroblastoma cell line	human	sCJD brain material	[119]
MDB	primary brain cells, SV40 transformed	mule deer	CWD	[129]
**2. Primary neuronal or brain-derived cells**				
CGN	cerebellar granule neurons overexpressing ovine PrP^C^	tgov mouse	mo 127S	[111]
CAS	cerebellar astrocytes overexpressing ovine PrP^C^	tgov mouse	mo 127S	[111]
NSC	neural stem cells	mouse	22L, RML	[112,113,115]
**3. Non-neuronal cells**				
C2C12	skeletal myoblast cell line	mouse	22L	[108]
L fibroblasts	fibroblast cell line	mouse	ME7, Chandler	[106]
L929	fibroblast cell line	mouse	22L, RML, ME7	[107]
NIH/3T3	fibroblast cell line	mouse	22L	[107]
MSC-80	Schwann cell line	mouse	Chandler	[156]
MovS	Schwann cell-like from dorsal root ganglia	tgov mouse	PG127, SSBP/1, scrapie field isolates	[104,157]
moRK13	epithelial cell line expressing mouse PrP^C^	rabbit	Fukuoka-1, 22L, Chandler, M1000, mo sCJD	[99,100,101,120]
voRK13	epithelial cell line expressing vole PrP^C^	rabbit	vo BSE	[100]
ovRK13/ RoV9	epithelial cell line expressing ovine PrP^C^	rabbit	PG127, LA404, SSBP/1, scrapie field isolates	[102,103,104]
elkRK13	epithelial cell line expressing elk PrP^C^	rabbit	CWD	[105,109]
**4. Primary non-neuronal cells**				
BM-derived MSC	bone marrow derived mesenchymal stem cell	mouse	Fukuoka-1	[110]
BM-derived MSC-like	bone marrow derived mesenchymal stem cell like	mouse	Fukuoka-1	[114]

* some cells overexpress moPrP^C^-A or 3F4 antibody-epitope tagged moPrP^C^

## 5. PrP^Sc^ Uptake During the Infection Process

The prion infection process *in vitro* can be divided into four main steps: 1) Attachment of PrP^Sc^ to the cell; 2) uptake; 3) initiation of PrP^Sc^ formation and establishment of productive infection; and 4) persistent propagation. Most of the steps have been studied separately. The use of different prion preparations, strains, and cell lines has complicated direct comparison of results. Consequently, the following paragraphs can only give an overview of the possible infection processes.

**Figure 3:**
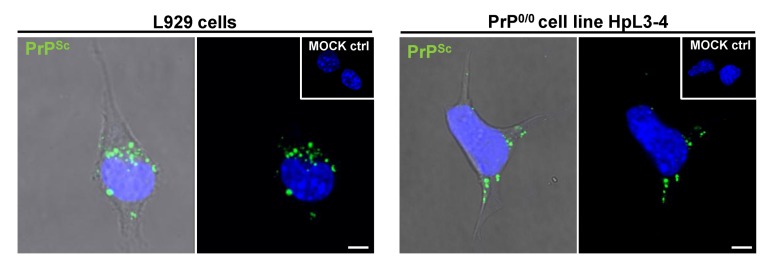
Non-neuronal cells and PrP-deficient cells take up PrP^Sc^. Brain homogenate from mice infected with the 22L prion strain is taken up by L929 fibroblast cells (left panel) and PrP-deficient HpL3-4 cells (right panel). Cells were incubated with infected brain homogenate for 18 hours prior to fixation, permeabilization, guanidine hydrochloride treatment and immunofluorescence staining. Cells incubated with uninfected brain homogenate (MOCK ctrl) served as control for specific detection of PrP^Sc^. PrP^Sc^ uptake is observed in both fibroblast cells and PrP-deficient cells. Monoclonal anti-PrP antibody: 4H11. Nuclei were counterstained with Hoechst (blue). Scale bar: 5 µm.

Most cell lines *in vitro* are capable of taking up PrP^Sc^ (Figure 3). Uptake of prion strains was reported to be neither cell type nor strain dependent [133]. However, even within a cell population exposed to scrapie brain homogenate, uptake is evident only in a subset of cells [133]. The observed differences in the speed of internalization are at least in part due to variations in the PrP^Sc^ sample preparation [133,158]. Detergent extraction of PrP^Sc^ prior to fluorescence labeling resulted in a slow uptake over a number of days [136]. However, PrP^Sc^ from crude brain homogenate preparations was taken up rapidly within minutes to hours post prion exposure [132,133,159,160,161,162,163,164]. Several studies have demonstrated that PrP^Sc^ is readily taken up by cells known to be resistant to prion infection [159,160,165,166], arguing that potential receptors and uptake mechanisms for PrP^Sc^ are also present in non-permissive cells.

As physical interaction between PrP^C^ and PrP^Sc^ is required for the conversion of cellular prion protein to its pathological isoform, PrP^C^ might also serve as a receptor for PrP^Sc^ uptake. Interestingly, overexpression of PrP^C^ did not affect initial binding of PrP^Sc^ to CHO cells [159]. It was later shown that cells devoid of PrP^C^ also take up PrP^Sc^, demonstrating that PrP^C^ is not generally required for PrP^Sc^ uptake (Figure 3) [133,136,159,162]. But how does PrP^Sc^ bind to the cell and how does it enter? Three putative cell surface receptors have been characterized that could be involved in PrP^Sc^ uptake. LRP/LR has been found expressed in human small intestinal mucosa [167], suggesting that it could mediate the initial PrP^Sc^ uptake in the gut when the animal is first exposed to prions by food contaminants. Importantly, PrP^Sc^ uptake in human intestinal enterocytes in culture depended on both prion preparations and strains [158]. Uptake of PrP^Sc^ present in brain homogenate from mice infected with bovine spongiform encephalopathy was reduced upon preincubation of cells with anti-LRP/LR antibodies, suggesting that LRP/LR is involved in this process. Likewise, uptake of proteinase K treated mouse-adapted scrapie prions into non-permissive BHK cells was dependent on the LRP/LR receptor and HS [166]. Of note, establishment of prion infection in these systems has not been shown.

Jen and colleagues recently demonstrated that a specific inhibitor of LRP1 receptors and siRNA-mediated knock-down both drastically impaired binding and uptake of both recombinant PrP fibrils and PrP^Sc^ in wildtype and PrP knock-out neurons [164]. Interestingly, addition of PrP^Sc^ to the cells slowed down endocytosis of endogenous PrP^C^, suggesting that PrP^Sc^ and PrP^C^ were competing for the same binding site on LRP1. Further studies demonstrated that the binding of PrP^Sc^ to LRP1 was mediated by cluster 4 of LRP1 that is also implicated in endocytosis of PrP^C^ [82,83,164].

Proteoglycans could represent the third class of PrP^Sc^ receptors necessary for binding and early uptake of exogenous PrP^Sc^. PrP^Sc^ binds to the HS analog heparin and disulfonated Congo red *in vitro* [159,168]. HS serves as a binding partner for PrP^Sc^* in vivo*, as proteinase K digested PrP^Sc^ (hamster scrapie Sc237) poorly bound to mutant CHO cells lacking HS or GAGs [159]. Addition of heparin, a natural HS analog, competitively inhibited binding of PrP^Sc^ to N2a and wildtype CHO cells [159]. GAG mimetics have also been shown to inhibit uptake of PrP^Sc^ in cell culture. Incubation of non-permissive CHO cells with heparan mimetic HM2602 drastically impaired uptake of hamster prion rods (strain Sc237) [165]. Likewise, DS500 and HM2602 impaired entry of hamster prion rods in N2a cells [160]. Of note, concentrations sufficient to inhibit PrP^Sc^ accumulation in RML infected N2a cells were inefficient in inhibiting PrP^Sc^ uptake [160]. A candidate proteoglycan for PrP^Sc^ binding and uptake is glypican-1 [169,170,171].

Although several putative receptors for PrP^Sc^ endocytosis have been identified, the exact mechanism of uptake has not been elucidated. Besides the classical endocytosis pathways of clathrin-mediated endocytosis or raft-mediated endocytosis, PrP^Sc^ could also be taken up by macropinocytosis. Macropinocytosis is a relatively non-selective process that delivers its cargo to late endosomal and lysosomal compartments. Studies on the uptake of fluorescently labeled detergent extracted, proteinase K treated PrP^Sc^ (Chandler scrapie strain) by SN56 cells revealed no co-localization with raft marker choleratoxin [136]. Instead, extensive co-localization was observed with fluorescent dextran, a marker for internalization by macropinocytosis. Addition of amiloride, an inhibitor of macropinocytosis, to Rov cells (RK13 cells expressing ovine PrP) did not inhibit uptake of exogenous PrP^Sc^, arguing that this internalization process is not involved in PrP^Sc^ uptake at least in these cells [162]. However, productive infection with RML prions was prevented in N2a cells upon addition of macropinocytosis inhibitor EIPA for 48 hours during the infection process [172]. Whether this treatment influenced external PrP^Sc^ uptake or impaired *de novo* PrP^Sc^ production has not been shown. Future studies will need to clarify the role of macropinocytosis for PrP^Sc^ uptake and establishment of persistent infections.

In summary, the mechanism of PrP^Sc^ internalization is not fully understood. PrP^Sc^ uptake might not be restricted to one pathway but could occur through multiple pathways [173] and host factors are likely to influence the outcome of the infection process [133,174,175]. One important question that needs to be addressed further is if the proposed uptake pathways also lead to a productive prion infection. So far, it cannot be excluded that productive infection requires a distinct internalization route and alternative uptake mechanisms might prevent chronic infection. Furthermore, it is unclear if different prion strains utilize the same entry pathways for establishing chronic infections.

## 6. Early Steps of Prion Infection

The aforementioned studies so far demonstrated that PrP^Sc^ can be taken up by a vast majority of cells *in vitro*, independent of PrP^C^ expression and receptors such as LRP/LR, LPR1 and proteoglycans might contribute to PrP^Sc^ internalization. But where exactly is PrP^Sc^ formed, and is the uptake of PrP^Sc^ necessary for a productive prion infection? Recent progress in studying the earliest events of prion infection has been made by expressing tagged PrP^C^ [132,134]. According to these studies, *de novo* PrP^Sc^ formation is a fast process, initiated within minutes [134] to hours post-exposure [132]. Remarkably, initial PrP^Sc^ formation was independent of the scrapie strain and was even apparent in cells that do not become persistently infected or with strains previously not shown to propagate in cell culture. However, PrP^Sc^ formation was often transient and did not result in a productive infection [132]. These data demonstrate that (1) non-permissive cells can transiently produce PrP^Sc^, (2) the establishment of a prion infection is initiated after the first round of PrP^Sc^ formation and (3) restricted susceptibility to certain strains is controlled by processes that take place after the initial PrP^Sc^ formation. Studies using myc-tagged PrP^C^ expressed in N2a cells demonstrated that PrP^Sc^ was formed on the plasma membrane within 2 minutes post prion exposure and was then rapidly trafficked to the perinuclear region [134]. Lipid rafts appeared to be important for PrP^Sc^ formation, as treatment with the cholesterol sequestering drug filipin, abolished this process [134]. Neither *de novo* PrP^Sc^ formation nor PrP^Sc^ accumulation in perinuclear compartments was abolished by inhibitors of dynamin-dependent endocytosis, CCPs or macropinocytosis. Thus, these endocytic pathways are either not involved in *de novo* formation and trafficking of PrP^Sc^ or multiple pathways can be utilized for PrP^Sc^ uptake [134]. The involvement of the LRP1 receptor for the establishment of a productive infection is unclear. Knock-down of LRP1 in sensory neurons during the acute infection step appeared to decrease uptake of PrP^Sc^ but had no influence on overall PrP^Sc^ levels four weeks post infection, a time point at which PrP^Sc^ replication is usually not observed in untreated sensory neurons [164]. Further experiments will be necessary to prove if the LRP1 receptor is also contributing to the establishment of a productive prion infection.

## 7. PrP^Sc^ Formation in Persistently Infected Cells

The cellular compartments involved in PrP^Sc^ formation and accumulation are still ill-defined. In cell culture, PrP^Sc^ accumulation has been reported mainly on the cell surface and within endocytic compartments [176,177,178], but also within vesicles of the secretory pathway [179,180,181], and even in the nucleus [182]. PrP^Sc^ formation is a post-translational event that requires physical interaction between PrP^Sc^ and PrP^C^ (Figure 2). Although both PrP^C^ and PrP^Sc^ are present on the plasma membrane of infected N2a cells [180,183], PrP^Sc^ localizes primarily intracellularly, with only minor amounts on the cell surface (Figure 1B) [131]. Still, transport of PrP^C^ to the plasma membrane is required for conversion into the abnormal isoform [92,134,177,178,181,184]. Removal of PrP^C^ from the plasma membrane by phospholipase C diminishes PrP^Sc^ accumulation in N2a cells [140,177,184]. Likewise, impaired transport of PrP^C^ to the cell surface by suramin cures chronically infected N2a cells and prevents PrP^Sc^ formation [92]. Lipid rafts appear to play an important role in the formation of PrP^Sc^ [58]. Detergent-resistant microdomains isolated from persistently infected N2a cells contain both PrP^C^ and PrP^Sc^ [25,179,181,184]. Inhibition of cellular cholesterol synthesis drastically impairs raft formation and also influences cellular PrP^Sc^ levels [24,179]. Filipin extraction of membrane cholesterol also affects cellular PrP^Sc^ levels in persistently infected N2a cells [65]. Mutant PrP^C^ with a transmembrane anchor that redistributes into non-raft regions is not converted to its abnormal isoform, suggesting that raft association is required for conversion. Of note, changing the PrP amino acid sequence by addition of a transmembrane anchor to PrP could also impair the conversion process *per se*, and the convertibility of such PrP molecule has not been formally proven *in vitro* [24]. Cells expressing PrP^C^ lacking the GPI moiety do not support sustained prion infection *in vitro*, arguing that the anchor is necessary for efficient PrP^Sc^ formation in cell culture [122].

The role of the secretory pathway for PrP^Sc^ formation is unclear. Early studies reported that PrP^Sc^ co-localized with Golgi markers [131]. It has been speculated that either PrP^C^ or PrP^Sc^ are directly translocated from the cell membrane to the ER by a Rab6 controlled retrograde pathway [185]. Interestingly, PrP mutants that are retained in the ER or Golgi apparatus can drastically interfere with PrP^Sc^ accumulation in RML infected N2a cells, suggesting that the mutants competitively inhibited binding or conversion of wildtype PrP^C^ in these compartments [186]. Alternatively, minute amounts of PrP^C^ trafficked correctly through the secretory pathway to the cell surface are capable of dominant negative interference with the conversion of PrP^C^.

An important role in the conversion of PrP^C^ to PrP^Sc ^in persistently infected cells is assigned to the endocytic pathway [176,177,178]. In primary hippocampal neurons, PrP^Sc^ was found at the cell surface and in early as well as recycling endosomes [61]. The early recycling compartment was suggested to be the primary location of prion conversion [187]. Recently, Zurzolo and co-workers studied the intracellular localization of PrP^Sc^ in three cell lines persistently infected with different prion strains and detected more than 25% of the protein co-localized with a marker for the early recycling compartment [187]. Others found that in chronically infected cell lines N2a and GT1, the majority of PrP^Sc^ accumulates intracellularly mainly localized within late endosomes and lysosomal compartments [131,177,181,184,188,189]. In endosomal or lysosomal compartments, PrP^Sc^ undergoes an initial proteolytic cleavage, leading to PrP^Sc^ lacking its amino-terminus [178,181,189,190]. Importantly, inhibition of amino-terminal trimming does not inhibit PrP^Sc^ accumulation, arguing that this step is not essential for PrP^Sc^ biogenesis [176,178]. In conclusion, while it is unclear if PrP^Sc^ replication mechanisms are the same for different strains and in different cell types, most studies argue that PrP^Sc^ formation in persistently infected cells takes place either on the cell surface or along the endocytic pathway, with the majority of PrP^Sc^ eventually accumulating in the lysosomal compartment (Figure 2B).

## 8. GAGs As Co-Factors for PrP^Sc^ Formation

The interaction of PrP^C^ and PrP^Sc^ with receptors for binding and uptake is closely linked to the conversion process. GAGs are not only involved in the binding and uptake of PrP, but also play an important role for PrP^Sc^ formation or stabilization. *In vivo* HS is a prominent component of cerebral prion amyloid plaques and diffuse PrP^Sc^ deposits [191]. Treatment of uninfected cells with lyases that cleave GAG chains from endogenous proteoglycans prevents prion infection, arguing that GAGs are essential for initiation of a productive prion infection [160]. However, GAGs also play an essential role in PrP^Sc^ accumulation in cells chronically infected with prions. Enzymatic digestion of cellular HS, but not cellular chondroitin or dermatan sulfate, reduced PrP^Sc^ levels in N2a cells chronically infected with RML prions, suggesting that HS is a major co-factor necessary during PrP^Sc^ biogenesis [91]. In line with this, sodium chlorate and xyloside EDX, inhibitors for sulfation and proteoglycan glycosylation, drastically reduced PrP^Sc^ levels in N2a cells chronically infected with RML [88,91]. Most exogenously added sulfated glycans interfere with PrP^Sc^ accumulation in a variety of persistently infected cell culture models, likely by binding to PrP^Sc^ or PrP^C^ and by competing for the interaction with endogenous sulfated glycans required for PrP^Sc^ formation and/or stabilization [88,89,192]. The degree of sulfation, but also other properties such as the glycan backbone, positioning of sulfates, non-sulfate substituents and glycan chain size are important for the anti-PrP^Sc^ activity of GAG analogs [89,165]. Disulfonated Congo red and sulfated glycans such as low molecular weight heparin, dextran sulfate, suramin and PPS all reduced PrP^Sc^ accumulation in N2a cells persistently infected with RML or Chandler [88,89,92,192]. Less sulfated HS, high molecular weight heparin, or other GAGs such as dermatan sulfate, chondroitin sulfate and hyaluronic acid exerted no anti-PrP^Sc^ activity [88]. HS side chains on glypican-1 are likely important for facilitating PrP conversion, as siRNA knock-down of glypican-1 significantly reduces total PrP^Sc^ levels in N2a cells [170]. In conclusion, *in vivo* and *in vitro* data argue that endogenous GAGs stimulate prion conversion, potentially by providing a scaffold for PrP^C^/PrP^Sc^ clustering and interaction [193,194,195]. Exogenous GAGs competitively inhibit the interaction of PrP^Sc^ and PrP^C^ with endogenous GAGs and thereby interfere with the conversion process.

## 9. Cell-To-Cell Transmission of Prions

Under the right culturing conditions, prion-infected cells retain stable PrP^Sc^ levels over multiple cell divisions. PrP^Sc^ accumulation in dividing cells is strongly influenced by the rate of PrP^Sc^ synthesis, degradation and cell division [196]. In persistently infected cells, prion infectivity is primarily transmitted from mother to daughter cells [196]. Interestingly, an increase of infected cells during cell propagation was observed in some [118,197] but not all cell cultures [107], arguing that at least in some cultures, prions spread to neighboring cells. Two major routes have been described for intercellular spread of prions *in vitro*. Several studies have reported release of PrP^Sc^ and/or infectivity into the cell culture medium (Table 2). Prions have also been found to be associated with exosomes released from infected cells [36,37,99,198]. In NIH/3T3 cells, retroviral co-infection enhanced the release of PrP^Sc^ and prion infectivity into the cell culture supernatant. Prion proteins were released in association with exosomes and viral particles, suggesting that retroviral co-infection could contribute to prion spreading [198]. Kanu and colleagues showed that in SMB cells infected with Chandler scrapie, cell-to-cell infection was dependent on close proximity or direct cell contact between donor and recipient cell [149]. Culturing infected and uninfected cell populations separated by transwells abolished infection of target cells. Likewise, conditioned medium was ineffective at transmitting prions to recipient cells. For some cell lines, secretion of infectivity has been reported, but prions were preferentially transmitted to nearby cells, suggesting that direct cell proximity promoted efficient infection [197]. The fact that living cells were far more effective in transmitting infectivity than dead cells argues that cell biological processes are involved in prion transmission. The exact mechanism of direct cell-to-cell spread in SMB, Mov and Rov cells needs to be determined, but recent studies argue that cytoplasmic bridges, so called tunneling nanotubes (TNTs), are involved in this process in CAD cells persistently infected with 139A prions [199]. TNTs are actin and/or microtubule containing cytoplasmic bridges that allow intercellular communication. These sometimes contradictory results might be explained by the use of different cell types and prion strains. Indeed, the intercellular transmission efficiency can differ significantly in different cell lines bearing comparable titers of the same prion strain, arguing that the ability to propagate and to disseminate prions are distinct phenomena [197].

**Table 2:** Routes of prion dissemination in cell culture.

Prion-infected donor cell line	Prion strain	Intercellular prion spreading	PrP^Sc^ secreted	References
N2a	22L	Yes, via conditioned medium	Yes, associated with exosomes	[36]
N2a	RML	No or inefficient	Not determined	[97,196]
SMB	Chandler	Yes, via direct cell contact	Not determined	[149]
HpL3-4*	22L	Yes, via conditioned medium	Not determined	[121]
NIH/3T3	22L	Yes, via conditioned medium	Yes, associated with exosomes	[198]
CAD	139A	Yes, via TNTs	Not determined	[200]
GT1	RML	Yes, via conditioned medium	Not determined	[97]
GT1	FU CJD	Yes, via conditioned medium	Not determined	[201]
GT1	M1000	Yes	Yes, associated with exosomes	[99]
ovRK13/ RoV9	PG127	Yes (inefficiently)	Yes, associated with exosomes	[37,197]
moRK13	M1000	Yes	Yes, associated with exosomes	[99]
Mov	PG127	Yes, via close proximity of cells	Yes, associated with exosomes	[37,111,197]
SN56	Chandler	Yes, via conditioned medium	Yes	[202]

* cells ectopically express 3F4 antibody-epitope tagged moPrP^C^

## 10. Other Protein Aggregates Can Spread and Propagate in Cell Culture

**Figure 4:**
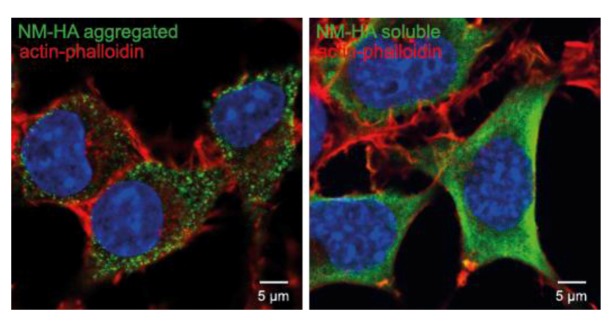
Propagation of cytosolic prions derived from the S. *cerevisiae* Sup35 prion domain NM. N2a cells ectopically express the HA-tagged prion domain NM of Sup35, which is the most well characterized yeast prion. The left image shows aggregated NM-HA (green) after induction with recombinant NM fibrils, the right image shows the soluble NM-HA (green). NM was stained with anti-HA antibody. F-Actin was stained with fluorescently conjugated phalloidin (red). Nuclei were stained with Hoechst (blue). Scale bar: 5 µm.

Over the last few years an increasing number of studies have shown that non-prion protein aggregates associated with other neurodegenerative diseases can spread from cell to cell in a prion-like manner [203]. The most studied amyloid proteins are Aβ and tau in Alzheimer`s disease (AD), α-synuclein in Parkinson`s disease (PD), superoxide dismutase 1 (SOD1) in amyotrophic lateral sclerosis (ALS), and polyglutamine-rich huntingtin fragments in Huntington`s disease (HD). These proteins differ from PrP in their amino acid sequences, functions and cellular locations, but all share the cross β-sheet conformation in their aggregated states. Although not infectious from a classical point of view, protein aggregates accumulating during those diseases have been shown to spread in tissues *in vivo* [204,205,206,207] and infect neighboring cells *in vitro* [208,209,210,211]. Spreading of protein misfolding along interconnected brain regions argues for direct cell contact as a potential route of transmission [212]. Co-cultures of donor and recipient cell lines demonstrated that tau, α-synuclein and SOD1 could be transmitted via conditioned medium, sometimes in association with exosomes [208,209,210,213,214]. A prerequisite of aggregate spreading is the presence of multiple seeds that can be transmitted in the infection process. The high spreading efficiency of prions compared to other amyloidogenic protein aggregates might, at least in part, be due to a more efficient aggregate fragmentation process that produces new seeds [203]. Using a model system of mammalian cells expressing the yeast prion protein Sup35 we have recently shown that the cytosol of mammalian cells provides an environment for efficient aggregate replication (Figure 4) [215]. The efficiency at which aggregate seeds are formed might differ depending on the protein aggregate, as Sup35 and SOD1 aggregates could be stably propagated over serial passages, while polyQ aggregates were diluted out over time [211,215,216].

## 11. Concluding Remarks

Almost 25 years after the discovery of prion susceptible neuroblastoma cells, persistently infected N2a cells still constitute the prototype cell-culture system for studying prions. Consequently, prion cell biology has been mostly studied in permanent cell lines chronically infected with prion strains RML, Chandler or 22L. Still, it is unclear if the identified pathways and co-factors are required for all prion strains, or if different prion strains utilize different subcellular compartments for efficient propagation. Clearly, pharmacological studies revealed significant differences in the anti-prion efficacy of compounds tested against various prion strains in permanent cells and primary neurons [111,217]. Whilst some of the anti-prion effects might be directly attributed to their special binding properties to PrP^C^ or PrP^Sc^ [193], some might exert their effect more indirectly by affecting cellular metabolism. Thus, thorough investigations with different strains propagated in the same cell line are necessary to determine if prion strains utilize the same cellular pathways and co-factors for initial infection and sustained propagation.

## References

[1] Beekes M., McBride P.A. (2007). The spread of prions through the body in naturally acquired transmissible spongiform encephalopathies. FEBS J..

[2] Bolton D.C., McKinley M.P., Prusiner S.B. (1982). Identification of a protein that purifies with the scrapie prion. Science.

[3] Prusiner S.B. (1982). Novel proteinaceous infectious particles cause scrapie. Science.

[4] Chiti F., Dobson C.M. (2006). Protein misfolding, functional amyloid, and human disease. Annu Rev. Biochem..

[5] Pattison I.H., Millson G.C. (1961). Scrapie produced experimentally in goats with special reference to the clinical syndrome. J. Comp. Pathol..

[6] Bessen R.A., Marsh R.F. (1992). Biochemical and physical properties of the prion protein from two strains of the transmissible mink encephalopathy agent. J. Virol..

[7] Safar J., Wille H., Itri V., Groth D., Serban H., Torchia M., Cohen F.E., Prusiner S.B. (1998). Eight prion strains have prp(sc) molecules with different conformations. Nat. Med..

[8] Asante E.A., Linehan J.M., Desbruslais M., Joiner S., Gowland I., Wood A.L., Welch J., Hill A.F., Lloyd S.E., Wadsworth J.D. (2002). Bse prions propagate as either variant cjd-like or sporadic cjd-like prion strains in transgenic mice expressing human prion protein. EMBO J..

[9] Cali I., Castellani R., Alshekhlee A., Cohen Y., Blevins J., Yuan J., Langeveld J.P., Parchi P., Safar J.G., Zou W.Q. (2009). Co-existence of scrapie prion protein types 1 and 2 in sporadic creutzfeldt-jakob disease: Its effect on the phenotype and prion-type characteristics. Brain.

[10] Weissmann C. (2005). Birth of a prion: Spontaneous generation revisited. Cell..

[11] Chesebro B., Race R., Wehrly K., Nishio J., Bloom M., Lechner D., Bergstrom S., Robbins K., Mayer L., Keith J.M. (1985). Identification of scrapie prion protein-specific mrna in scrapie-infected and uninfected brain. Nature.

[12] Basler K., Oesch B., Scott M., Westaway D., Walchli M., Groth D.F., McKinley M.P., Prusiner S.B., Weissmann C. (1986). Scrapie and cellular prp isoforms are encoded by the same chromosomal gene. Cell..

[13] Robakis N.K., Sawh P.R., Wolfe G.C., Rubenstein R., Carp R.I., Innis M.A. (1986). Isolation of a cdna clone encoding the leader peptide of prion protein and expression of the homologous gene in various tissues. Proc. Natl. Acad. Sci. USA.

[14] Sparkes R.S., Simon M., Cohn V.H., Fournier R.E., Lem J., Klisak I., Heinzmann C., Blatt C., Lucero M., Mohandas T. (1986). Assignment of the human and mouse prion protein genes to homologous chromosomes. Proc. Natl. Acad. Sci. USA.

[15] Oesch B., Westaway D., Prusiner S.B. (1991). Prion protein genes: Evolutionary and functional aspects. Curr. Top. Microbiol. Immunol..

[16] Schatzl H.M., Da Costa M., Taylor L., Cohen F.E., Prusiner S.B. (1995). Prion protein gene variation among primates. J. Mol. Biol.

[17] Wopfner F., Weidenhofer G., Schneider R., von Brunn A., Gilch S., Schwarz T.F., Werner T., Schatzl H.M. (1999). Analysis of 27 mammalian and 9 avian prps reveals high conservation of flexible regions of the prion protein. J. Mol. Biol..

[18] Linden R., Martins V.R., Prado M.A., Cammarota M., Izquierdo I., Brentani R.R. (2008). Physiology of the prion protein. Physiol. Rev..

[19] Brown D.R., Schmidt B., Kretzschmar H.A. (1996). Role of microglia and host prion protein in neurotoxicity of a prion protein fragment. Nature.

[20] Moser M., Colello R.J., Pott U., Oesch B. (1995). Developmental expression of the prion protein gene in glial cells. Neuron.

[21] Haraguchi T., Fisher S., Olofsson S., Endo T., Groth D., Tarentino A., Borchelt D.R., Teplow D., Hood L., Burlingame A. (1989). Asparagine-linked glycosylation of the scrapie and cellular prion proteins. Arch. Biochem. Biophys..

[22] Turk E., Teplow D.B., Hood L.E., Prusiner S.B. (1988). Purification and properties of the cellular and scrapie hamster prion proteins. Eur. J. Biochem..

[23] Stahl N., Baldwin M.A., Hecker R., Pan K.M., Burlingame A.L., Prusiner S.B. (1992). Glycosylinositol phospholipid anchors of the scrapie and cellular prion proteins contain sialic acid. Biochemistry.

[24] Taraboulos A., Scott M., Semenov A., Avrahami D., Laszlo L., Prusiner S.B. (1995). Cholesterol depletion and modification of cooh-terminal targeting sequence of the prion protein inhibit formation of the scrapie isoform. J. Cell. Biol.

[25] Vey M., Pilkuhn S., Wille H., Nixon R., DeArmond S.J., Smart E.J., Anderson R.G., Taraboulos A., Prusiner S.B. (1996). Subcellular colocalization of the cellular and scrapie prion proteins in caveolae-like membranous domains. Proc. Natl. Acad. Sci. USA.

[26] Baron G.S., Wehrly K., Dorward D.W., Chesebro B., Caughey B. (2002). Conversion of raft associated prion protein to the protease-resistant state requires insertion of prp-res (prp(sc)) into contiguous membranes. EMBO J..

[27] Walmsley A.R., Watt N.T., Taylor D.R., Perera W.S., Hooper N.M. (2009). Alpha-cleavage of the prion protein occurs in a late compartment of the secretory pathway and is independent of lipid rafts. Mol. Cell. Neurosci..

[28] Sarnataro D., Campana V., Paladino S., Stornaiuolo M., Nitsch L., Zurzolo C. (2004). Prp(c) association with lipid rafts in the early secretory pathway stabilizes its cellular conformation. Mol. Biol. Cell..

[29] Mironov A., Latawiec D., Wille H., Bouzamondo-Bernstein E., Legname G., Williamson R.A., Burton D., DeArmond S.J., Prusiner S.B., Peters P.J. (2003). Cytosolic prion protein in neurons. J. Neurosci..

[30] Vincent B., Paitel E., Frobert Y., Lehmann S., Grassi J., Checler F. (2000). Phorbol ester-regulated cleavage of normal prion protein in hek293 human cells and murine neurons. J. Biol. Chem..

[31] Vincent B., Paitel E., Saftig P., Frobert Y., Hartmann D., De Strooper B., Grassi J., Lopez-Perez E., Checler F. (2001). The disintegrins adam10 and tace contribute to the constitutive and phorbol ester-regulated normal cleavage of the cellular prion protein. J. Biol. Chem..

[32] Parkin E.T., Watt N.T., Turner A.J., Hooper N.M. (2004). Dual mechanisms for shedding of the cellular prion protein. J. Biol. Chem..

[33] Alfa Cisse M., Sunyach C., Slack B.E., Fisher A., Vincent B., Checler F. (2007). M1 and m3 muscarinic receptors control physiological processing of cellular prion by modulating adam17 phosphorylation and activity. J. Neurosci..

[34] Borchelt D.R., Rogers M., Stahl N., Telling G., Prusiner S.B. (1993). Release of the cellular prion protein from cultured cells after loss of its glycoinositol phospholipid anchor. Glycobiology.

[35] Starke R., Harrison P., Drummond O., Macgregor I., Mackie I., Machin S. (2003). The majority of cellular prion protein released from endothelial cells is soluble. Transfusion.

[36] Alais S., Simoes S., Baas D., Lehmann S., Raposo G., Darlix J.L., Leblanc P. (2008). Mouse neuroblastoma cells release prion infectivity associated with exosomal vesicles. Biol. Cell.

[37] Fevrier B., Vilette D., Archer F., Loew D., Faigle W., Vidal M., Laude H., Raposo G. (2004). Cells release prions in association with exosomes. Proc. Natl. Acad. Sci. USA.

[38] Rane N.S., Yonkovich J.L., Hegde R.S. (2004). Protection from cytosolic prion protein toxicity by modulation of protein translocation. EMBO J..

[39] Crozet C., Vezilier J., Delfieu V., Nishimura T., Onodera T., Casanova D., Lehmann S., Beranger F. (2006). The truncated 23–230 form of the prion protein localizes to the nuclei of inducible cell lines independently of its nuclear localization signals and is not cytotoxic. Mol. Cell. Neurosci..

[40] Linden R., Cordeiro Y., Lima L.M. (2012). Allosteric function and dysfunction of the prion protein. Cell. Mol. Life Sci..

[41] Gibbings D., Leblanc P., Jay F., Pontier D., Michel F., Schwab Y., Alais S., Lagrange T., Voinnet O. (2012). Human prion protein binds argonaute and promotes accumulation of microrna effector complexes. Nat. Struct. Mol. Biol..

[42] Cheng F., Lindqvist J., Haigh C.L., Brown D.R., Mani K. (2006). Copper-dependent co-internalization of the prion protein and glypican-1. J. Neurochem..

[43] Watt N.T., Taylor D.R., Kerrigan T.L., Griffiths H.H., Rushworth J.V., Whitehouse I.J., Hooper N.M. (2012). Prion protein facilitates uptake of zinc into neuronal cells. Nat. Commun..

[44] Graner E., Mercadante A.F., Zanata S.M., Forlenza O.V., Cabral A.L., Veiga S.S., Juliano M.A., Roesler R., Walz R., Minetti A. (2000). Cellular prion protein binds laminin and mediates neuritogenesis. Brain Res. Mol..

[45] Zanata S.M., Lopes M.H., Mercadante A.F., Hajj G.N., Chiarini L.B., Nomizo R., Freitas A.R., Cabral A.L., Lee K.S., Juliano M.A. (2002). Stress-inducible protein 1 is a cell surface ligand for cellular prion that triggers neuroprotection. EMBO J..

[46] Santuccione A., Sytnyk V., Leshchyns'ka I., Schachner M. (2005). Prion protein recruits its neuronal receptor ncam to lipid rafts to activate p59fyn and to enhance neurite outgrowth. J. Cell. Biol..

[47] Zhang C.C., Steele A.D., Lindquist S., Lodish H.F. (2006). Prion protein is expressed on long-term repopulating hematopoietic stem cells and is important for their self-renewal. Proc. Natl. Acad. Sci. USA.

[48] Steele A.D., Emsley J.G., Ozdinler P.H., Lindquist S., Macklis J.D. (2006). Prion protein (prpc) positively regulates neural precursor proliferation during developmental and adult mammalian neurogenesis. Proc. Natl. Acad. Sci. USA.

[49] Gabus C., Auxilien S., Pechoux C., Dormont D., Swietnicki W., Morillas M., Surewicz W., Nandi P., Darlix J.L. (2001). The prion protein has DNA strand transfer properties similar to retroviral nucleocapsid protein. J. Mol. Biol..

[50] Gabus C., Derrington E., Leblanc P., Chnaiderman J., Dormont D., Swietnicki W., Morillas M., Surewicz W.K., Marc D., Nandi P. (2001). The prion protein has rna binding and chaperoning properties characteristic of nucleocapsid protein ncp7 of hiv-1. J. Biol. Chem..

[51] Cordeiro Y., Machado F., Juliano L., Juliano M.A., Brentani R.R., Foguel D., Silva J.L. (2001). DNA converts cellular prion protein into the beta-sheet conformation and inhibits prion peptide aggregation. J. Biol. Chem..

[52] Lima L.M., Cordeiro Y., Tinoco L.W., Marques A.F., Oliveira C.L., Sampath S., Kodali R., Choi G., Foguel D., Torriani I. (2006). Structural insights into the interaction between prion protein and nucleic acid. Biochemistry.

[53] Morris R.J., Parkyn C.J., Jen A. (2006). Traffic of prion protein between different compartments on the neuronal surface, and the propagation of prion disease. FEBS Lett..

[54] Sunyach C., Jen A., Deng J., Fitzgerald K.T., Frobert Y., Grassi J., McCaffrey M.W., Morris R. (2003). The mechanism of internalization of glycosylphosphatidylinositol-anchored prion protein. EMBO J..

[55] Caetano F.A., Lopes M.H., Hajj G.N., Machado C.F., Pinto Arantes C., Magalhaes A.C., Vieira Mde P., Americo T.A., Massensini A.R., Priola S.A. (2008). Endocytosis of prion protein is required for erk1/2 signaling induced by stress-inducible protein 1. J. Neurosci.

[56] Magalhaes A.C., Silva J.A., Lee K.S., Martins V.R., Prado V.F., Ferguson S.S., Gomez M.V., Brentani R.R., Prado M.A. (2002). Endocytic intermediates involved with the intracellular trafficking of a fluorescent cellular prion protein. J. Biol. Chem..

[57] Prado M.A., Alves-Silva J., Magalhaes A.C., Prado V.F., Linden R., Martins V.R., Brentani R.R. (2004). Prpc on the road: Trafficking of the cellular prion protein. J. Neurochem..

[58] Campana V., Sarnataro D., Zurzolo C. (2005). The highways and byways of prion protein trafficking. Trends Cell. Biol..

[59] Stuermer C.A., Langhorst M.F., Wiechers M.F., Legler D.F., Von Hanwehr S.H., Guse A.H., Plattner H. (2004). Prpc capping in t cells promotes its association with the lipid raft proteins reggie-1 and reggie-2 and leads to signal transduction. FASEB J..

[60] Shyng S.L., Huber M.T., Harris D.A. (1993). A prion protein cycles between the cell surface and an endocytic compartment in cultured neuroblastoma cells. J. Biol. Chem..

[61] Godsave S.F., Wille H., Kujala P., Latawiec D., DeArmond S.J., Serban A., Prusiner S.B., Peters P.J. (2008). Cryo-immunogold electron microscopy for prions: Toward identification of a conversion site. J. Neurosci..

[62] Peters P.J., Mironov A.., Peretz D., van Donselaar E., Leclerc E., Erpel S., DeArmond S.J., Burton D.R., Williamson R.A., Vey M. (2003). Trafficking of prion proteins through a caveolae-mediated endosomal pathway. J. Cell. Biol..

[63] Baumann M.H., Kallijarvi J., Lankinen H., Soto C., Haltia M. (2000). Apolipoprotein e includes a binding site which is recognized by several amyloidogenic polypeptides. Biochem. J..

[64] Kang Y.S., Zhao X., Lovaas J., Eisenberg E., Greene L.E. (2009). Clathrin-independent internalization of normal cellular prion protein in neuroblastoma cells is associated with the arf6 pathway. J. Cell. Sci..

[65] Marella M., Lehmann S., Grassi J., Chabry J. (2002). Filipin prevents pathological prion protein accumulation by reducing endocytosis and inducing cellular prp release. J. Biol. Chem..

[66] Taylor D.R., Watt N.T., Perera W.S., Hooper N.M. (2005). Assigning functions to distinct regions of the n-terminus of the prion protein that are involved in its copper-stimulated, clathrin-dependent endocytosis. J. Cell Sci..

[67] Watt N.T., Hooper N.M. (2005). Reactive oxygen species (ros)-mediated beta-cleavage of the prion protein in the mechanism of the cellular response to oxidative stress. Biochem. Soc. Trans..

[68] Martins V.R., Graner E., Garcia-Abreu J., de Souza S.J., Mercadante A.F., Veiga S.S., Zanata S.M., Neto V.M., Brentani R.R. (1997). Complementary hydropathy identifies a cellular prion protein receptor. Nat. Med..

[69] Pauly P.C., Harris D.A. (1998). Copper stimulates endocytosis of the prion protein. J. Biol. Chem..

[70] Madore N., Smith K.L., Graham C.H., Jen A., Brady K., Hall S., Morris R. (1999). Functionally different gpi proteins are organized in different domains on the neuronal surface. EMBO J..

[71] Galvan C., Camoletto P.G., Dotti C.G., Aguzzi A., Ledesma M.D. (2005). Proper axonal distribution of prp(c) depends on cholesterol-sphingomyelin-enriched membrane domains and is developmentally regulated in hippocampal neurons. Mol. Cell. Neurosci..

[72] Sarnataro D., Caputo A., Casanova P., Puri C., Paladino S., Tivodar S.S., Campana V., Tacchetti C., Zurzolo C. (2009). Lipid rafts and clathrin cooperate in the internalization of prp in epithelial frt cells. PLoS One.

[73] Kirchhausen T. (2000). Clathrin. Annu Rev. Biochem..

[74] Gauczynski S., Peyrin J.M., Haik S., Leucht C., Hundt C., Rieger R., Krasemann S., Deslys J.P., Dormont D., Lasmezas C.I. (2001). The 37-kda/67-kda laminin receptor acts as the cell-surface receptor for the cellular prion protein. EMBO J..

[75] Gauczynski S., Hundt C., Leucht C., Weiss S. (2001). Interaction of prion proteins with cell surface receptors, molecular chaperones, and other molecules. Adv. Protein. Chem..

[76] Rieger R., Edenhofer F., Lasmezas C.I., Weiss S. (1997). The human 37-kda laminin receptor precursor interacts with the prion protein in eukaryotic cells. Nat. Med..

[77] Hundt C., Peyrin J.M., Haik S., Gauczynski S., Leucht C., Rieger R., Riley M.L., Deslys J.P., Dormont D., Lasmezas C.I. (2001). Identification of interaction domains of the prion protein with its 37-kda/67-kda laminin receptor. EMBO J..

[78] Douville P.J., Harvey W.J., Carbonetto S. (1988). Isolation and partial characterization of high affinity laminin receptors in neural cells. J. Biol. Chem..

[79] Watts J.C., Huo H., Bai Y., Ehsani S., Jeon A.H., Shi T., Daude N., Lau A., Young R., Xu L. (2009). Interactome analyses identify ties of prp and its mammalian paralogs to oligomannosidic n-glycans and endoplasmic reticulum-derived chaperones. PLoS Pathog..

[80] Nykjaer A., Willnow T.E. (2002). The low-density lipoprotein receptor gene family: A cellular swiss army knife?. Trends Cell. Biol..

[81] Wu L., Gonias S.L. (2005). The low-density lipoprotein receptor-related protein-1 associates transiently with lipid rafts. J. Cell. Biochem..

[82] Taylor D.R., Hooper N.M. (2007). The low-density lipoprotein receptor-related protein 1 (lrp1) mediates the endocytosis of the cellular prion protein. Biochem. J..

[83] Parkyn C.J., Vermeulen E.G., Mootoosamy R.C., Sunyach C., Jacobsen C., Oxvig C., Moestrup S., Liu Q., Bu G., Jen A. (2008). Lrp1 controls biosynthetic and endocytic trafficking of neuronal prion protein. J. Cell. Sci..

[84] Wang S., Herndon M.E., Ranganathan S., Godyna S., Lawler J., Argraves W.S., Liau G. (2004). Internalization but not binding of thrombospondin-1 to low density lipoprotein receptor-related protein-1 requires heparan sulfate proteoglycans. J. Cell. Biochem..

[85] Yin S., Yu S., Li C., Wong P., Chang B., Xiao F., Kang S.C., Yan H., Xiao G., Grassi J. (2006). Prion proteins with insertion mutations have altered n-terminal conformation and increased ligand binding activity and are more susceptible to oxidative attack. J. Biol. Chem..

[86] Pan T., Wong B.S., Liu T., Li R., Petersen R.B., Sy M.S. (2002). Cell-surface prion protein interacts with glycosaminoglycans. Biochem. J..

[87] Warner R.G., Hundt C., Weiss S., Turnbull J.E. (2002). Identification of the heparan sulfate binding sites in the cellular prion protein. J. Biol. Chem..

[88] Gabizon R., Meiner Z., Halimi M., Ben-Sasson S.A. (1993). Heparin-like molecules bind differentially to prion-proteins and change their intracellular metabolic fate. J. Cell. Physiol..

[89] Caughey B., Raymond G.J. (1993). Sulfated polyanion inhibition of scrapie-associated prp accumulation in cultured cells. J. Virol..

[90] Shyng S.L., Lehmann S., Moulder K.L., Harris D.A. (1995). Sulfated glycans stimulate endocytosis of the cellular isoform of the prion protein, prpc, in cultured cells. J. Biol. Chem..

[91] Ben-Zaken O., Tzaban S., Tal Y., Horonchik L., Esko J.D., Vlodavsky I., Taraboulos A. (2003). Cellular heparan sulfate participates in the metabolism of prions. J. Biol. Chem..

[92] Gilch S., Winklhofer K.F., Groschup M.H., Nunziante M., Lucassen R., Spielhaupter C., Muranyi W., Riesner D., Tatzelt J., Schatzl H.M. (2001). Intracellular re-routing of prion protein prevents propagation of prp(sc) and delays onset of prion disease. EMBO J..

[93] Clarke M.C., Haig D.A. (1970). Multiplication of scrapie agent in cell culture. Res. Vet. Sci.

[94] Caughey B., Race R.E., Ernst D., Buchmeier M.J., Chesebro B. (1989). Prion protein biosynthesis in scrapie-infected and uninfected neuroblastoma cells. J. Virol..

[95] Butler D.A., Scott M.R., Bockman J.M., Borchelt D.R., Taraboulos A., Hsiao K.K., Kingsbury D.T., Prusiner S.B. (1988). Scrapie-infected murine neuroblastoma cells produce protease-resistant prion proteins. J. Virol.

[96] Nishida N., Harris D.A., Vilette D., Laude H., Frobert Y., Grassi J., Casanova D., Milhavet O., Lehmann S. (2000). Successful transmission of three mouse-adapted scrapie strains to murine neuroblastoma cell lines overexpressing wild-type mouse prion protein. J. Virol..

[97] Schatzl H.M., Laszlo L., Holtzman D.M., Tatzelt J., DeArmond S.J., Weiner R.I., Mobley W.C., Prusiner S.B. (1997). A hypothalamic neuronal cell line persistently infected with scrapie prions exhibits apoptosis. J. Virol..

[98] Iwamaru Y., Takenouchi T., Ogihara K., Hoshino M., Takata M., Imamura M., Tagawa Y., Hayashi-Kato H., Ushiki-Kaku Y., Shimizu Y. (2007). Microglial cell line established from prion protein-overexpressing mice is susceptible to various murine prion strains. J. Virol..

[99] Vella L.J., Sharples R.A., Lawson V.A., Masters C.L., Cappai R., Hill A.F. (2007). Packaging of prions into exosomes is associated with a novel pathway of prp processing. J. Pathol..

[100] Courageot M.P., Daude N., Nonno R., Paquet S., Di Bari M.A., Le Dur A., Chapuis J., Hill A.F., Agrimi U., Laude H. (2008). A cell line infectible by prion strains from different species. J. Gen. Virol..

[101] Lawson V.A., Vella L.J., Stewart J.D., Sharples R.A., Klemm H., Machalek D.M., Masters C.L., Cappai R., Collins S.J., Hill A.F. (2008). Mouse-adapted sporadic human creutzfeldt-jakob disease prions propagate in cell culture. Int. J. Biochem. Cell. Biol..

[102] Vilette D., Andreoletti O., Archer F., Madelaine M.F., Vilotte J.L., Lehmann S., Laude H. (2001). *Ex vivo* propagation of infectious sheep scrapie agent in heterologous epithelial cells expressing ovine prion protein. Proc. Natl. Acad. Sci. USA.

[103] Sabuncu E., Petit S., Le Dur A., Lan Lai T., Vilotte J.L., Laude H., Vilette D. (2003). Prp polymorphisms tightly control sheep prion replication in cultured cells. J. Virol..

[104] Neale M.H., Mountjoy S.J., Edwards J.C., Vilette D., Laude H., Windl O., Saunders G.C. (2010). Infection of cell lines with experimental and natural ovine scrapie agents. J. Virol..

[105] Bian J., Napier D., Khaychuck V., Angers R., Graham C., Telling G. (2010). Cell-based quantification of chronic wasting disease prions. J. Virol..

[106] Clarke M.C., Millson G.C. (1976). Infection of a cell line of mouse l fibroblasts with scrapie agent. Nature.

[107] Vorberg I., Raines A., Story B., Priola S.A. (2004). Susceptibility of common fibroblast cell lines to transmissible spongiform encephalopathy agents. J. Infect. Dis..

[108] Dlakic W.M., Grigg E., Bessen R.A. (2007). Prion infection of muscle cells *in vitro*. J. Virol..

[109] Kim H.J., Tark D.S., Lee Y.H., Kim M.J., Lee W.Y., Cho I.S., Sohn H.J., Yokoyama T. (2012). Establishment of a cell line persistently infected with chronic wasting disease prions. J. Vet. Med. Sci..

[110] Akimov S., Vasilyeva I., Yakovleva O., McKenzie C., Cervenakova L. (2009). Murine bone marrow stromal cell culture with features of mesenchymal stem cells susceptible to mouse-adapted human tse agent, fukuoka-1. Folia. Neuropathol..

[111] Cronier S., Laude H., Peyrin J.M. (2004). Prions can infect primary cultured neurons and astrocytes and promote neuronal cell death. Proc. Natl. Acad. Sci. USA.

[112] Giri R.K., Young R., Pitstick R., DeArmond S.J., Prusiner S.B., Carlson G.A. (2006). Prion infection of mouse neurospheres. Proc. Natl. Acad. Sci. USA.

[113] Milhavet O., Casanova D., Chevallier N., McKay R.D., Lehmann S. (2006). Neural stem cell model for prion propagation. Stem. Cell..

[114] Cervenakova L., Akimov S., Vasilyeva I., Yakovleva O., McKenzie C., Cervenak J., Piccardo P., Asher D.M. (2011). Fukuoka-1 strain of transmissible spongiform encephalopathy agent infects murine bone marrow-derived cells with features of mesenchymal stem cells. Transfusion.

[115] Herva M.E., Relano-Gines A., Villa A., Torres J.M. (2010). Prion infection of differentiated neurospheres. J. Neurosci. Meth..

[116] Polymenidou M., Trusheim H., Stallmach L., Moos R., Julius C., Miele G., Lenz-Bauer C., Aguzzi A. (2008). Canine mdck cell lines are refractory to infection with human and mouse prions. Vaccine.

[117] Gibson P.E., Bell T.M., Field E.J. (1972). Failure of the scrapie agent to replicate in l5178y mouse leukaemic cells. Res. Vet. Sci..

[118] Klohn P.C., Stoltze L., Flechsig E., Enari M., Weissmann C. (2003). A quantitative, highly sensitive cell-based infectivity assay for mouse scrapie prions. Proc. Natl. Acad. Sci. USA.

[119] Ladogana A., Liu Q., Xi Y.G., Pocchiari M. (1995). Proteinase-resistant protein in human neuroblastoma cells infected with brain material from creutzfeldt-jakob patient. Lancet.

[120] Lewis V., Hill A.F., Haigh C.L., Klug G.M., Masters C.L., Lawson V.A., Collins S.J. (2009). Increased proportions of c1 truncated prion protein protect against cellular m1000 prion infection. J. Neuropathol. Exp. Neurol..

[121] Maas E., Geissen M., Groschup M.H., Rost R., Onodera T., Schatzl H., Vorberg I.M. (2007). Scrapie infection of prion protein-deficient cell line upon ectopic expression of mutant prion proteins. J. Biol. Chem..

[122] McNally K.L., Ward A.E., Priola S.A. (2009). Cells expressing anchorless prion protein are resistant to scrapie infection. J. Virol..

[123] Bosque P.J., Ryou C., Telling G., Peretz D., Legname G., DeArmond S.J., Prusiner S.B. (2002). Prions in skeletal muscle. Proc. Natl. Acad. Sci. USA.

[124] Bosque P.J., Prusiner S.B. (2000). Cultured cell sublines highly susceptible to prion infection. J. Virol..

[125] Mahal S.P., Baker C.A., Demczyk C.A., Smith E.W., Julius C., Weissmann C. (2007). Prion strain discrimination in cell culture: The cell panel assay. Proc. Natl. Acad. Sci. USA.

[126] Bach C., Gilch S., Rost R., Greenwood A.D., Horsch M., Hajj G.N., Brodesser S., Facius A., Schadler S., Sandhoff K. (2009). Prion-induced activation of cholesterogenic gene expression by srebp2 in neuronal cells. J. Biol. Chem..

[127] Race R.E., Fadness L.H., Chesebro B. (1987). Characterization of scrapie infection in mouse neuroblastoma cells. J. Gen. Virol.

[128] Kocisko D.A., Caughey B. (2006). Searching for anti-prion compounds: Cell-based high-throughput *in vitro* assays and animal testing strategies. Methods Enzymol..

[129] Raymond G.J., Olsen E.A., Lee K.S., Raymond L.D., Bryant P.K., Baron G.S., Caughey W.S., Kocisko D.A., McHolland L.E., Favara C. (2006). Inhibition of protease-resistant prion protein formation in a transformed deer cell line infected with chronic wasting disease. J. Virol..

[130] Chasseigneaux S., Pastore M., Britton-Davidian J., Manie E., Stern M.H., Callebert J., Catalan J., Casanova D., Belondrade M., Provansal M. (2008). Genetic heterogeneity *versus *molecular analysis of prion susceptibility in neuroblasma n2a sublines. Arch. Virol..

[131] Taraboulos A., Serban D., Prusiner S.B. (1990). Scrapie prion proteins accumulate in the cytoplasm of persistently infected cultured cells. J. Cell. Biol..

[132] Vorberg I., Raines A., Priola S.A. (2004). Acute formation of protease-resistant prion protein does not always lead to persistent scrapie infection *in vitro*. J. Biol. Chem..

[133] Greil C.S., Vorberg I.M., Ward A.E., Meade-White K.D., Harris D.A., Priola S.A. (2008). Acute cellular uptake of abnormal prion protein is cell type and scrapie-strain independent. Virology.

[134] Goold R., Rabbanian S., Sutton L., Andre R., Arora P., Moonga J., Clarke A.R., Schiavo G., Jat P., Collinge J. (2011). Rapid cell-surface prion protein conversion revealed using a novel cell system. Nat. Commun..

[135] Bian J., Nazor K.E., Angers R., Jernigan M., Seward T., Centers A., Green M., Telling G.C. (2006). Gfp-tagged prp supports compromised prion replication in transgenic mice. Biochem. Biophys. Res. Comm..

[136] Magalhaes A.C., Baron G.S., Lee K.S., Steele-Mortimer O., Dorward D., Prado M.A., Caughey B. (2005). Uptake and neuritic transport of scrapie prion protein coincident with infection of neuronal cells. J. Neurosci..

[137] Mahal S.P., Demczyk C.A., Smith E.W., Klohn P.C., Weissmann C. (2008). Assaying prions in cell culture: The standard scrapie cell assay (ssca) and the scrapie cell assay in end point format (scepa). Methods Mol. Biol..

[138] Arellano-Anaya Z.E., Savistchenko J., Mathey J., Huor A., Lacroux C., Andreoletti O., Vilette D. (2011). A simple, versatile and sensitive cell-based assay for prions from various species. PLoS One.

[139] Arjona A., Simarro L., Islinger F., Nishida N., Manuelidis L. (2004). Two creutzfeldt-jakob disease agents reproduce prion protein-independent identities in cell cultures. Proc. Natl. Acad. Sci. USA.

[140] Enari M., Flechsig E., Weissmann C. (2001). Scrapie prion protein accumulation by scrapie-infected neuroblastoma cells abrogated by exposure to a prion protein antibody. Proc. Natl. Acad. Sci. USA.

[141] Markovits P., Dautheville C., Dormont D., Dianoux L., Latarjet R. (1983). *In vitro* propagation of the scrapie agent. I. Transformation of mouse glia and neuroblastoma cells after infection with the mouse-adapted scrapie strain c-506. Acta. Neuropathol..

[142] Ostlund P., Lindegren H., Pettersson C., Bedecs K. (2001). Up-regulation of functionally impaired insulin-like growth factor-1 receptor in scrapie-infected neuroblastoma cells. J. Biol. Chem..

[143] Race R. (1991). The scrapie agent *in vitro*. Curr Top. Microbiol. Immunol..

[144] Scott M.R., Kohler R., Foster D., Prusiner S.B. (1992). Chimeric prion protein expression in cultured cells and transgenic mice. Protein. Sci..

[145] Miyazawa K., Emmerling K., Manuelidis L. (2011). High cjd infectivity remains after prion protein is destroyed. J. Cell. Biochem..

[146] Baron T.G., Biacabe A.G., Bencsik A., Langeveld J.P. (2006). Transmission of new bovine prion to mice. Emerg. Infect. Dis..

[147] Nunziante M., Ackermann K., Dietrich K., Wolf H., Gadtke L., Gilch S., Vorberg I., Groschup M., Schatzl H.M. (2011). Proteasomal dysfunction and endoplasmic reticulum stress enhance trafficking of prion protein aggregates through the secretory pathway and increase accumulation of pathologic prion protein. J. Biol. Chem..

[148] Birkett C.R., Hennion R.M., Bembridge D.A., Clarke M.C., Chree A., Bruce M.E., Bostock C.J. (2001). Scrapie strains maintain biological phenotypes on propagation in a cell line in culture. EMBO J..

[149] Kanu N., Imokawa Y., Drechsel D.N., Williamson R.A., Birkett C.R., Bostock C.J., Brockes J.P. (2002). Transfer of scrapie prion infectivity by cell contact in culture. Curr. Biol..

[150] Dron M., Dandoy-Dron F., Farooq Salamat M.K., Laude H. (2009). Proteasome inhibitors promote the sequestration of prpsc into aggresomes within the cytosol of prion-infected cad neuronal cells. J. Gen. Virol..

[151] Browning S., Baker C.A., Smith E., Mahal S.P., Herva M.E., Demczyk C.A., Li J., Weissmann C. (2011). Abrogation of complex glycosylation by swainsonine results in strain- and cell-specific inhibition of prion replication. J. Biol. Chem..

[152] Julius C., Hutter G., Wagner U., Seeger H., Kana V., Kranich J., Klohn P.C., Weissmann C., Miele G., Aguzzi A. (2008). Transcriptional stability of cultured cells upon prion infection. J. Mol. Biol..

[153] Rubenstein R., Deng H., Race R.E., Ju W., Scalici C.L., Papini M.C., Kascsak R.J., Carp R.I. (1992). Demonstration of scrapie strain diversity in infected pc12 cells. J. Gen. Virol.

[154] Rubenstein R., Carp R.I., Callahan S.M. (1984). *In vitro* replication of scrapie agent in a neuronal model: Infection of pc12 cells. J. Gen. Virol..

[155] Rubenstein R., Deng H., Scalici C.L., Papini M.C. (1991). Alterations in neurotransmitter-related enzyme activity in scrapie-infected pc12 cells. J. Gen. Virol..

[156] Follet J., Lemaire-Vieille C., Blanquet-Grossard F., Podevin-Dimster V., Lehmann S., Chauvin J.P., Decavel J.P., Varea R., Grassi J., Fontes M. (2002). Prp expression and replication by schwann cells: Implications in prion spreading. J. Virol..

[157] Archer F., Bachelin C., Andreoletti O., Besnard N., Perrot G., Langevin C., Le Dur A., Vilette D., Baron-Van Evercooren A., Vilotte J.L. (2004). Cultured peripheral neuroglial cells are highly permissive to sheep prion infection. J. Virol..

[158] Morel E., Andrieu T., Casagrande F., Gauczynski S., Weiss S., Grassi J., Rousset M., Dormont D., Chambaz J. (2005). Bovine prion is endocytosed by human enterocytes via the 37 kda/67 kda laminin receptor. Am. J. Pathol..

[159] Hijazi N., Kariv-Inbal Z., Gasset M., Gabizon R. (2005). Prpsc incorporation to cells requires endogenous glycosaminoglycan expression. J. Biol. Chem..

[160] Horonchik L., Tzaban S., Ben-Zaken O., Yedidia Y., Rouvinski A., Papy-Garcia D., Barritault D., Vlodavsky I., Taraboulos A. (2005). Heparan sulfate is a cellular receptor for purified infectious prions. J. Biol. Chem..

[161] Mohan J., Hopkins J., Mabbott N.A. (2005). Skin-derived dendritic cells acquire and degrade the scrapie agent following *in vitro* exposure. Immunology.

[162] Paquet S., Daude N., Courageot M.P., Chapuis J., Laude H., Vilette D. (2007). Prpc does not mediate internalization of prpsc but is required at an early stage for de novo prion infection of rov cells. J. Virol..

[163] Langevin C., Gousset K., Costanzo M., Richard-Le Goff O., Zurzolo C. (2010). Characterization of the role of dendritic cells in prion transfer to primary neurons. Biochem. J..

[164] Jen A., Parkyn C.J., Mootoosamy R.C., Ford M.J., Warley A., Liu Q., Bu G., Baskakov I.V., Moestrup S., McGuinness L. (2010). Neuronal low-density lipoprotein receptor-related protein 1 binds and endocytoses prion fibrils via receptor cluster 4. J. Cell. Sci..

[165] Schonberger O., Horonchik L., Gabizon R., Papy-Garcia D., Barritault D., Taraboulos A. (2003). Novel heparan mimetics potently inhibit the scrapie prion protein and its endocytosis. Biochem. Biophys. Res. Commun..

[166] Gauczynski S., Nikles D., El-Gogo S., Papy-Garcia D., Rey C., Alban S., Barritault D., Lasmezas C.I., Weiss S. (2006). The 37-kda/67-kda laminin receptor acts as a receptor for infectious prions and is inhibited by polysulfated glycanes. J. Infect. Dis..

[167] Shmakov A.N., Bode J., Kilshaw P.J., Ghosh S. (2000). Diverse patterns of expression of the 67-kd laminin receptor in human small intestinal mucosa: Potential binding sites for prion proteins?. J. Pathol..

[168] Prusiner S.B., McKinley M.P., Bowman K.A., Bolton D.C., Bendheim P.E., Groth D.F., Glenner G.G. (1983). Scrapie prions aggregate to form amyloid-like birefringent rods. Cell.

[169] Lofgren K., Wahlstrom A., Lundberg P., Langel U., Graslund A., Bedecs K. (2008). Antiprion properties of prion protein-derived cell-penetrating peptides. FASEB J..

[170] Taylor D.R., Whitehouse I.J., Hooper N.M. (2009). Glypican-1 mediates both prion protein lipid raft association and disease isoform formation. PLoS Pathog..

[171] Hooper N.M. (2011). Glypican-1 facilitates prion conversion in lipid rafts. J. Neurochem..

[172] Wadia J.S., Schaller M., Williamson R.A., Dowdy S.F. (2008). Pathologic prion protein infects cells by lipid-raft dependent macropinocytosis. PLoS One.

[173] Taylor D.R., Hooper N.M. (2006). The prion protein and lipid rafts. Mol. Membr. Biol..

[174] Deleault N.R., Harris B.T., Rees J.R., Supattapone S. (2007). Formation of native prions from minimal components *in vitro*. Proc. Natl. Acad. Sci. USA.

[175] Abid K., Morales R., Soto C. (2010). Cellular factors implicated in prion replication. FEBS Lett..

[176] Caughey B., Raymond G.J., Ernst D., Race R.E. (1991). N-terminal truncation of the scrapie-associated form of prp by lysosomal protease(s): Implications regarding the site of conversion of prp to the protease-resistant state. J. Virol..

[177] Borchelt D.R., Taraboulos A., Prusiner S.B. (1992). Evidence for synthesis of scrapie prion proteins in the endocytic pathway. J. Biol. Chem..

[178] Taraboulos A., Raeber A.J., Borchelt D.R., Serban D., Prusiner S.B. (1992). Synthesis and trafficking of prion proteins in cultured cells. Mol. Biol. Cell..

[179] Naslavsky N., Stein R., Yanai A., Friedlander G., Taraboulos A. (1997). Characterization of detergent-insoluble complexes containing the cellular prion protein and its scrapie isoform. J. Biol. Chem..

[180] Stahl N., Baldwin M.A., Burlingame A.L., Prusiner S.B. (1990). Identification of glycoinositol phospholipid linked and truncated forms of the scrapie prion protein. Biochemistry.

[181] Veith N.M., Plattner H., Stuermer C.A., Schulz-Schaeffer W.J., Burkle A. (2009). Immunolocalisation of prpsc in scrapie-infected n2a mouse neuroblastoma cells by light and electron microscopy. Eur. J. Cell Biol..

[182] Mange A., Crozet C., Lehmann S., Beranger F. (2004). Scrapie-like prion protein is translocated to the nuclei of infected cells independently of proteasome inhibition and interacts with chromatin. J. Cell Sci..

[183] Lehmann S., Harris D.A. (1996). Mutant and infectious prion proteins display common biochemical properties in cultured cells. J. Biol. Chem..

[184] Caughey B., Raymond G.J. (1991). The scrapie-associated form of prp is made from a cell surface precursor that is both protease- and phospholipase-sensitive. J. Biol. Chem..

[185] Beranger F., Mange A., Goud B., Lehmann S. (2002). Stimulation of prp(c) retrograde transport toward the endoplasmic reticulum increases accumulation of prp(sc) in prion-infected cells. J. Biol. Chem..

[186] Vorberg I., Chan K., Priola S.A. (2001). Deletion of beta-strand and alpha-helix secondary structure in normal prion protein inhibits formation of its protease-resistant isoform. J. Virol..

[187] Marijanovic Z., Caputo A., Campana V., Zurzolo C. (2009). Identification of an intracellular site of prion conversion. PLoS Pathog..

[188] McKinley M.P., Taraboulos A., Kenaga L., Serban D., Stieber A., DeArmond S.J., Prusiner S.B., Gonatas N. (1991). Ultrastructural localization of scrapie prion proteins in cytoplasmic vesicles of infected cultured cells. Lab. Invest..

[189] Pimpinelli F., Lehmann S., Maridonneau-Parini I. (2005). The scrapie prion protein is present in flotillin-1-positive vesicles in central- but not peripheral-derived neuronal cell lines. Eur. J. Neurosci..

[190] McKinley M.P., Meyer R.K., Kenaga L., Rahbar F., Cotter R., Serban A., Prusiner S.B. (1991). Scrapie prion rod formation *in vitro* requires both detergent extraction and limited proteolysis. J. Virol..

[191] Snow A.D., Kisilevsky R., Willmer J., Prusiner S.B., DeArmond S.J. (1989). Sulfated glycosaminoglycans in amyloid plaques of prion diseases. Acta. Neuropathol..

[192] Adjou K.T., Simoneau S., Sales N., Lamoury F., Dormont D., Papy-Garcia D., Barritault D., Deslys J.P., Lasmezas C.I. (2003). A novel generation of heparan sulfate mimetics for the treatment of prion diseases. J. Gen. Virol..

[193] Wong C., Xiong L.W., Horiuchi M., Raymond L., Wehrly K., Chesebro B., Caughey B. (2001). Sulfated glycans and elevated temperature stimulate prp(sc)-dependent cell-free formation of protease-resistant prion protein. EMBO J..

[194] Murayama Y., Yoshioka M., Masujin K., Okada H., Iwamaru Y., Imamura M., Matsuura Y., Fukuda S., Onoe S., Yokoyama T. (2010). Sulfated dextrans enhance *in vitro* amplification of bovine spongiform encephalopathy prp(sc) and enable ultrasensitive detection of bovine prp(sc). PLoS One.

[195] Yokoyama T., Takeuchi A., Yamamoto M., Kitamoto T., Ironside J.W., Morita M. (2011). Heparin enhances the cell-protein misfolding cyclic amplification efficiency of variant creutzfeldt-jakob disease. Neurosci. Lett..

[196] Ghaemmaghami S., Phuan P.W., Perkins B., Ullman J., May B.C., Cohen F.E., Prusiner S.B. (2007). Cell division modulates prion accumulation in cultured cells. Proc. Natl. Acad. Sci. USA.

[197] Paquet S., Langevin C., Chapuis J., Jackson G.S., Laude H., Vilette D. (2007). Efficient dissemination of prions through preferential transmission to nearby cells. J. Gen. Virol..

[198] Leblanc P., Alais S., Porto-Carreiro I., Lehmann S., Grassi J., Raposo G., Darlix J.L. (2006). Retrovirus infection strongly enhances scrapie infectivity release in cell culture. EMBO J..

[199] Gousset K., Zurzolo C. (2009). Tunnelling nanotubes: A highway for prion spreading?. Prion.

[200] Gousset K., Schiff E., Langevin C., Marijanovic Z., Caputo A., Browman D.T., Chenouard N., de Chaumont F., Martino A., Enninga J. (2009). Prions hijack tunnelling nanotubes for intercellular spread. Nat. Cell. Biol..

[201] Nishida N., Katamine S., Manuelidis L. (2005). Reciprocal interference between specific cjd and scrapie agents in neural cell cultures. Science.

[202] Baron G.S., Magalhaes A.C., Prado M.A., Caughey B. (2006). Mouse-adapted scrapie infection of sn56 cells: Greater efficiency with microsome-associated *versus *purified prp-res. J. Virol..

[203] Krammer C., Schatzl H.M., Vorberg I. (2009). Prion-like propagation of cytosolic protein aggregates: Insights from cell culture models. Prion.

[204] Luk K.C., Kehm V.M., Zhang B., O'Brien P., Trojanowski J.Q., Lee V.M. (2012). Intracerebral inoculation of pathological alpha-synuclein initiates a rapidly progressive neurodegenerative alpha-synucleinopathy in mice. J. Exp. Med..

[205] Meyer-Luehmann M., Coomaraswamy J., Bolmont T., Kaeser S., Schaefer C., Kilger E., Neuenschwander A., Abramowski D., Frey P., Jaton A.L. (2006). Exogenous induction of cerebral beta-amyloidogenesis is governed by agent and host. Science.

[206] Desplats P., Lee H.J., Bae E.J., Patrick C., Rockenstein E., Crews L., Spencer B., Masliah E., Lee S.J. (2009). Inclusion formation and neuronal cell death through neuron-to-neuron transmission of alpha-synuclein. Proc. Natl. Acad. Sci. USA.

[207] Clavaguera F., Bolmont T., Crowther R.A., Abramowski D., Frank S., Probst A., Fraser G., Stalder A.K., Beibel M., Staufenbiel M. (2009). Transmission and spreading of tauopathy in transgenic mouse brain. Nat. Cell. Biol.

[208] Munch C., O'Brien J., Bertolotti A. (2011). Prion-like propagation of mutant superoxide dismutase-1 misfolding in neuronal cells. Proc. Natl. Acad. Sci. USA.

[209] Kfoury N., Holmes B.B., Jiang H., Holtzman D.M., Diamond M.I. (2012). Trans-cellular propagation of tau aggregation by fibrillar species. J. Biol. Chem..

[210] Hansen C., Angot E., Bergstrom A.L., Steiner J.A., Pieri L., Paul G., Outeiro T.F., Melki R., Kallunki P., Fog K. (2011). Alpha-synuclein propagates from mouse brain to grafted dopaminergic neurons and seeds aggregation in cultured human cells. J. Clin. Invest..

[211] Ren P.H., Lauckner J.E., Kachirskaia I., Heuser J.E., Melki R., Kopito R.R. (2009). Cytoplasmic penetration and persistent infection of mammalian cells by polyglutamine aggregates. Nat. Cell. Biol..

[212] Braak H., Del Tredici K., Rub U., de Vos R.A., Jansen Steur E.N., Braak E. (2003). Staging of brain pathology related to sporadic parkinson's disease. Neurobiol. Aging.

[213] Lee H.J., Patel S., Lee S.J. (2005). Intravesicular localization and exocytosis of alpha-synuclein and its aggregates. J. Neurosci..

[214] Luk K.C., Kehm V., Carroll J., Zhang B., O'Brien P., Trojanowski J.Q., Lee V.M. (2012). Pathological alpha-synuclein transmission initiates parkinson-like neurodegeneration in nontransgenic mice. Science.

[215] Krammer C., Kryndushkin D., Suhre M.H., Kremmer E., Hofmann A., Pfeifer A., Scheibel T., Wickner R.B., Schatzl H.M., Vorberg I. (2009). The yeast sup35nm domain propagates as a prion in mammalian cells. Proc. Natl. Acad. Sci. USA.

[216] Munch C., Bertolotti A. (2011). Self-propagation and transmission of misfolded mutant sod1: Prion or prion-like phenomenon?. Cell. Cycle..

[217] Kocisko D.A., Engel A.L., Harbuck K., Arnold K.M., Olsen E.A., Raymond L.D., Vilette D., Caughey B. (2005). Comparison of protease-resistant prion protein inhibitors in cell cultures infected with two strains of mouse and sheep scrapie. Neurosci. Lett..

